# Stabilization of lead in incineration fly ash by moderate thermal treatment with sodium hydroxide addition

**DOI:** 10.1371/journal.pone.0178816

**Published:** 2017-06-06

**Authors:** Bing Gong, Yi Deng, Yuanyi Yang, Yong He, Xiaolong Sun, Li-Ya Ge, Kewei Zhang, Weizhong Yang

**Affiliations:** 1 School of Materials Science and Engineering and School of Chemical Engineering, Sichuan University, Chengdu, China; 2 Department of Materials Engineering, Sichuan College of Architectural Technology, Deyang, China; 3 CECEP Chengdu Renewable Energy Co. Ltd., Chengdu, China; 4 Zerowaste Asia Co. Ltd., Singapore, Singapore; The Education University of Hong Kong, HONG KONG

## Abstract

Municipal solid waste (MSW) incineration fly ash (IFA) can be potentially reused as a substitute for some raw materials, but treatment for detoxification is indispensable owing to high contents of heavy metals in fly ash. In the present work, due to excessive leaching concentration of lead (Pb), a moderate thermal treatment with sodium hydroxide (NaOH) addition was employed to stabilize Pb in IFA. The moderate thermal treatment was performed under relatively low temperature ranging from 300 to 500°C and at retention time from 1 to 3 h with NaOH addition in a range of 1 to 9%. Leaching results showed that leaching concentrations of Pb in IFA leachates decreased below the standard for hazardous waste identification (5 mg/L) in China under all treatment scenarios. With the increase of temperature, retention time and the amount of NaOH, the concentration of Pb were further suppressed in IFA leachates. Especially, at 500°C for 3 h with 9% NaOH addition, the concentration of Pb dropped to 0.18 mg/L, which was below the standard for sanitary landfill (0.25 mg/L) in China. In thermal process, needle-like crystallites melted to form granules and clumps with compacter structure and less pores. After the thermal treatment, water-soluble and exchangeable fractions of Pb significantly decreased. Therefore, the thermal treatment coupled with NaOH could be applied to realize the environmentally sound management of MSW IFA.

## Introduction

With the flourishing development of global economy and urbanization, a large quantity of municipal solid waste (MSW) and its sharp increase have been two of the most critical issues worldwide. It was reported that the amount of MSW was soaring at a rate of 2 billion tons annually [1]. The existing landfills tend to be fully filled and there is little land space for new sites. Moreover, it is widely confirmed that the disposal of MSW in landfills has induced sever deterioration of air quality, water quality and public health and so on [2, 3]. As a consequence, the incineration of MSW is of great benefits for waste management, which has been strongly encouraged. For example, the incineration can not only reduce the volume and mass of wastes, but also realize energy recovery and disinfection. The procedure of MSW combustion generates residues, which are prevailingly divided into incineration fly ash (IFA) and incineration bottom ash (IBA) [4, 5]. Generally, IBA is not regarded as a hazardous residue due to its low content of detrimental substance, and is extensively employed as an auxiliary construction material in many countries (e.g. Germany) [6]. Conversely, IFA is classified as a hazardous by-product because it contains multitudes of heavy metals like Cd, Zn, Pb, Hg, Cu, Cr, etc and the potential leachability of these heavy metals is beyond the regulations in most cases [7]. There is no doubt that immediate reuse or disposal in landfills of IFA is not permitted and treatment for detoxicating IFA is in high demand [8, 9].

Washing process can separate a large proportion of heavy metals and soluble salts from IFA using water or acid aqueous solution. Water washing is obviously effective for reducing the concentrations of chloride and heavy metals in IFA; however, its effectiveness for detoxification is limited and can hardly meet the relevant regulations. With regard to acid washing, previous literatures demonstrated that it was more efficient for the removal of heavy metals from IFA compared with water washing. Irrespective of water or acid washing, the generated waste solution has abundant heavy metals and soluble salts, which could be a potential problem of serious secondary pollution. In addition, consumption of large quantity of fresh water and costly acid is never advisable [10, 11].

Stabilization/solidification (S/S) treatments are the more extensive techniques for IFA detoxification which astrict the leaching of heavy metals in IFA by forming steady blocks or inertial compounds with additives or binders [12, 13]. S/S methods can be divided into solidification, chemical stabilization, and weathering [11]. Solidification, namely encapsulation, is a process to mix IFA with liquid (water or aqueous solution) firstly and then to solidify the slurry. Solids obtained from this process are less toxic than the raw IFA, and are more desirable for landfills or reutilization. On the other hand, the objective of chemical stabilization is to attain chemically inert materials with or without solidification. However, there are some shortcomings, which should be concerned. Long-term immobility of chemically treated residues and high cost of expensive reagents or binders cannot be ignored. Moreover, increment in the mass and volume of residue caused by the use of binders in S/S process may hinder its widespread application [14]. Weathering method involves a long processing period even using acceleration technologies [15], and its availability for some metals like Cu is not satisfactory [16].

Thermal treatment was considered as a promising method to immobilize heavy metals *via* heating IFA at very high temperature in a range of 700 to 1600°C [17–20]. Thermal techniques, including vitrification, sintering, and melting, solidified residues with a notable volume reduction. Products from the procedures are extremely stable, and leaching concentrations of heavy metals are far under regulations [20–22]. Nevertheless, several fatal defects deserve intense attention: (1) Huge consumption of energy is noncompliance with the trend of energy saving worldwide; (2) High cost of the approach evokes great concern as well [23, 24]; (3) Some heavy metals with comparatively low volatility like Cr, Cu, Pb, and Zn can be easily vaporized giving rise to secondary environmental contamination [25, 26]. On the basis of the above facts, the utilization of conventional thermal treatment should be deliberately assessed.

In the present work, the IFA samples were treated under relatively low temperature ranging from 300 to 500°C. The influences of temperature, retention time and the amount of sodium hydroxide (NaOH) addition were systematically investigated. Leaching tests of nascent and thermal treated IFA were conducted to evaluate the efficiency of the approach. The difference in distribution of Pb speciation of raw and treated IFA was revealed with sequential extraction experiment. For heavy metals in raw IFA, Pb leaching concentration merely exceeded the limits according to environmental protection law of China, and thus we only focused on the leaching behavior of Pb before and after the thermal treatment. The originality of this study was to stabilize Pb *via* thermal process under relatively low temperature using NaOH as an additive. NaOH has quite low melting point (318°C), which implies that liquid phase is easy to form during thermal process. This property facilitates chemical reactions and structure densification which contributes to the stabilization of Pb in IFA. From another aspect, NaOH is extremely prone to absorb moisture from air and reacts with carbon dioxide (CO_2_). Thus, hydrothermal solidification and carbonation in local areas could occur in heating process, which was conducive to immobilize the heavy metals in IFA. Simultaneously, thermal process in a lower temperature than general temperature (above 700°C) tremendously reduced energy consumption. Therefore, this moderate thermal treatment coupled with NaOH addition could be recommended for MSW IFA detoxification.

## Materials and methods

### 2.1. Materials

The pristine IFA in this study was sampled from a full-scale operating plant with an air pollution control (APC) system in Chengdu China, which can handle 1800 tons MSW per day. In the APC system, lime mortar was injected into flue gas to absorb components like HCl, etc.

The raw samples were first collected from the IFA silo of MSW incineration plant and then dried to constant weight at 105°C ±0.5°C for 24 h. After that, the dried IFA was sealed in a polyethylene drum for subsequent experiments. All staring materials derived from the same batch. Granular NaOH of analytical reagent grade (mass content ≥ 98.0%) was purchased from Chengdu KeLong reagent Co., Ltd.

### 2.2. Experimental procedure

**Fig 1:**
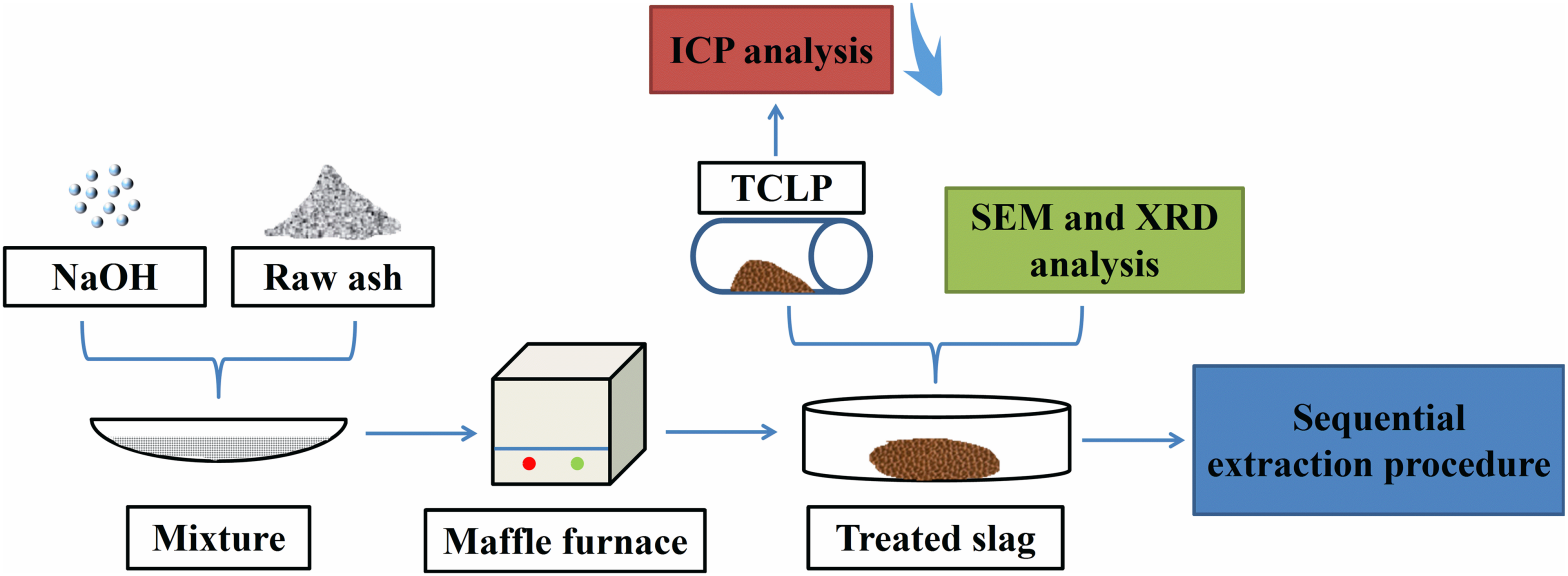
The experimental scheme of thermal treatment process.

Table 1 lists the detailed experimental conditions, and Fig 1 depicts experimental process of thermal treatment in the present study. The effects of temperature and retention time were investigated with 9% NaOH addition, and then the impact of different amounts of NaOH was discussed under the optimized set of temperature and retention time. Initially, IFA samples were weighed and mixed with defined amount of NaOH, afterwards the mixtures were pushed into a muffle furnace (SGM. 08/15A, Luoyang Sigma Furnace Industry Co., Ltd) and heated to targeted temperature. After held for a preset period, the residues were naturally cooled down to ambient temperature for succedent analysis.

**Table 1:** Operating conditions of the experiment.

Sample name	Targeted temperature (°C)	Retention time (h)	Amount of NaOH (%)
R319	300	1	9
R329	300	2	9
R339	300	3	9
R419	400	1	9
R429	400	2	9
R439	400	3	9
R519	500	1	9
R529	500	2	9
R539	500	3	9
R530	500	3	0
R531	500	3	1
R533	500	3	3
R535	500	3	5
R537	500	3	7

According to Chinese standard (HJ/T 300–2007) [27], the leaching tests using acetic acid buffer solution (pH = 2.64 ± 0.05), before and after thermal treatment, were performed to determine the leaching concentration of Pb. The extraction solution was prepared by diluting 17.25 mL glacial acetic acid with 1 L of deionized water. In the leaching procedure, 10 g of solid samples and 200 mL (L/S = 20) were blended in a 2 L polyethylene bottle. The mixture was then agitated in an end-over-end tumbler for 18 h ± 2 h at a speed of 30 ± 2 rpm. After the agitation, the suspension was filtered through 0.45-μm membrane disk filter and then injected into 15-mL polyethylene tube. All filtered samples were acidified to pH < 2 using nitric acid and preserved at 4°C prior to chemical analysis. Finally, the raw IFA and residue treated at 500°C for 3 h with 9% NaOH addition were subject to sequential extraction procedure described in Section 3.6.

### 2.3. Characterization and analyses

#### 2.3.1. Particle size distribution

The particle size distribution analysis of raw FA was conducted using laser diffraction particle size analyzer (LPSA; JL-1177, JNGX, China) with deionized water as dispersing media. The concentration was approximately 0.1 mg solid/mL water. The raw IFA was mixed with deionized water in a glass beaker and then the suspension was subject to ultrasonic dispersion before test.

#### 2.3.2. Chemical components

Main chemical components of the starting IFA were determined by X-ray fluorescence (XRF) spectrometry (Shimadzu sequential XRF-1800, Shimadzu, Japan) which can determine Elements from ^4^Be to ^92^U with Rh-anti-cathode, 4KW thin window, the working voltage of 40kV, and the current of 40mA. The analysis progress lasted for 10 min.

#### 2.3.3. Heavy metal leachability

Leaching concentrations of heavy metals in different filtered leachates were determined using inductively coupled plasma-atomic emission spectroscopy (ICP-AES; IRIS Advantage, Thermo Jarrell Aha Corporation, America). The detection limits of the method for heavy metals of Cd, Cr, Cu, Pb, and Zn were 0.0007 mg/L, 0.0066 mg/L, 0.002 mg/L, 0.002 mg/L, 0.005mg/L, respectively. The concentration of Hg in the leachates of raw IFA was detected with an inductively coupled plasma-mass spectrometer (ICP-MS; PQ ExCell, Themo Elemental, America) and the detection limit for Hg was 0.0001 mg/L.

#### 2.3.4. Mineralogy

Principal crystalline phase analysis was implemented by X-ray powder diffraction (XRD; LabX XRD-6100, Shimadzu, Japan) with Cu-Kα radiation (λ = 0.15418 nm) and scanning ranging from 10 to 80° with 0.02°/step each lasting 1.2 s. The working voltage was 40 kV and the current was 30 mA.

#### 2.3.5. Morphology

Scanning electron microscope (SEM; JSM-5900LV, JEOL, Japan) equipped with an energy dispersive X-ray spectrometer (EDS; INCA 250, Oxford Instruments, UK) was employed to observe morphology characteristics of the IFA particles and detect elemental compositions of local area on the individual particles surface with a typical accelerating voltage of 15kV. Samples were coated with gold using a sputter coater.

## Results and discussion

### 3.1. Characteristics of raw IFA

**Fig 2:**
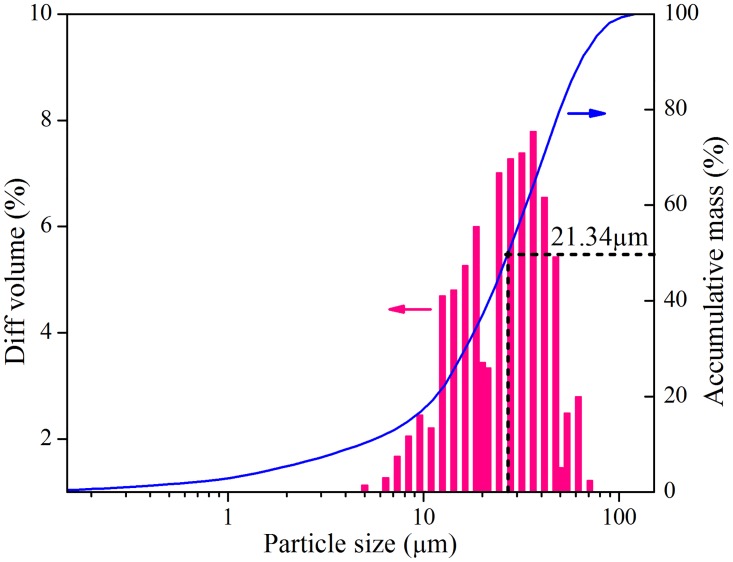
Particle size distribution of raw IFA.

Particle size distribution of raw IFA is presented in Fig 2. The average D(50) particle size (50% of the particle sizes are smaller than this value) was 21.34 μm. Table 2 displays main chemical compositions of raw IFA determined by XRF. Calcium oxide (CaO) and chlorine accounted for 46.6% and 21.1% in IFA, respectively. The vast-scale injection of lime mortar for air pollution control was responsible for high content of CaO in IFA, and alkaline condition was beneficial for heavy metal immobilization. High content of chlorine derived from the combustion of wood, kitchen garbage and PVC, which were abundant in the input MSW. Chlorine commonly combined with sodium and potassium in the form of NaCl and KCl. On the other hand, huge numbers of heavy metals in input MSW and their evaporation during MSW incineration accounted for high contents of heavy metals in fresh ash. Lastly, loss on ignition (LOI) of fresh ash was 10.5% which was caused by water evaporation, organic matters volatilization, and carbonates decomposition.

**Table 2:** Basic compositions of the raw IFA determined from XRF.

Compositions	The raw IFA (%)
SiO_2_	4.78
CaO	46.55
Al_2_O_3_	1.12
SO_3_	7.31
P_2_O_5_	0.5
Fe_2_O_3_	0.92
MgO	1.63
Na_2_O	9.01
Cl	21.11
K_2_O	5.7
TiO_2_	0.34
Zn(mg/kg)	3611.1
Cu(mg/kg)	624
Cr (mg/kg)	259.7
Pb(mg/kg)	1754.8
LOI	10.5%

Table 3 reveals the leaching concentrations of several heavy metals in raw IFA and corresponding limits required in the identification standard for hazardous wastes (GB 5085.3–2007, labeled as S1) and the standard for pollution control on the security landfill site (GB 16889–2008, labeled as S2). As shown in Table 3, leaching concentrations of Cd, Cr, Cu, and Zn were far below both limits, and that of Hg agreed with the two standards as well. However, leaching concentration of Pb was about 4 fold higher than the limit in S1 and 77 times higher than the regulation in S2. Therefore, it was imperative to decrease leaching concentration of Pb in raw IFA before its recovery or end in sanitary landfills. In the following sections, focus point of this study would be only the stabilization of Pb in IFA.

**Table 3:** Leaching concentrations of heavy metals in IFA and their limits in S1 and S2.

Heavy metals	Cd	Cr	Cu	Pb	Zn	Hg
Leaching data (mg/L)	0.0026	0.1541	0.1121	19.27	1.126	0.037
Limits in S1	1	5	100	5	100	0.1
Limits in S2	0.15	1.5	40	0.25	100	0.05

### 3.2. Leaching results of Pb in thermal treated IFAs

**Fig 3:**
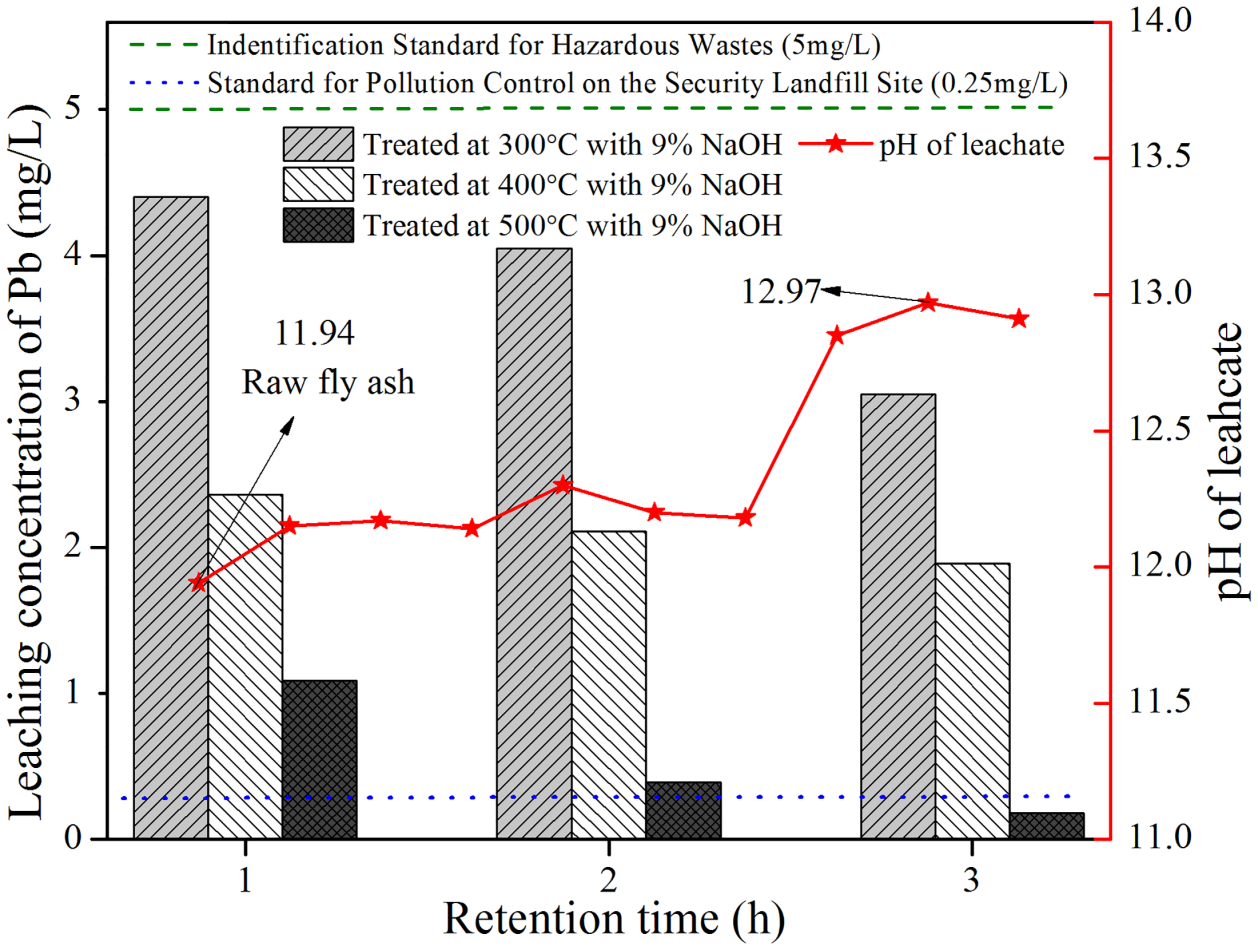
TCLP leaching concentrations of Pb in different treated residues.

The efficiency of thermal treatment under different temperatures and retention times with 9% NaOH addition was verified by TCLP leaching test. Fig 3 depicts the pH values of leachate and leaching concentrations of Pb from treated slags. Compared with leaching concentration of Pb from raw IFA (19.27 mg/L), leaching concentrations of Pb from all thermal treated products decreased sharply, and were below the limitation regulated in S1 (5 mg/L), which meant that all detoxified residues were not hazardous wastes. As shown in the Fig 3, with the extension of duration and experimental temperature, Pb was markedly less leachable. Especially for R539, leaching concentration of Pb was 0.18 mg/L which was below the regulation in S2 (0.25 mg/L), indicating that the product of R539 could be managed in sanitary landfill site.

At the preliminary stage of thermal process, particles contacted with each other in a “point contact mode”. However, as heating proceeded, the connection area between adjacent particles extended and particles combined more firmly, and the number of open pores declined dramatically [17, 28]. After thermal treatment, compact particle clusters came into being. As a result, leaching behavior of Pb in thermal treated products was significantly suppressed. In addition, longer duration and higher temperature facilitated structure densification. This also partly interpreted that leaching concentrations of Pb from residues treated at 300°C for 1 h were much higher than IFA treated at 400°C and 500°C for 3 h. It was worthy of mentioning that the property of low melting point of NaOH (318°C) made it more easily and efficiently form aggregated particles. Besides, it is well accepted that NaOH is extremely liable to absorb moisture and reacts with carbon dioxide in air. Thus, hydrothermal solidification and carbonation occurred in microcell, and newly-formed minerals like carbonates and silicates covered the surface indicating that encapsulation and adsorption impeded the leaching of Pb. It is exhibited in Fig 3 and S1 Table that pH values of thermal treated products range from 12.12 to 12.97. In the range of 300 to 400°C, pH of leachate remained steady, and was only slightly higher than that of raw ash. However, when temperature was enhanced to 500°C, pH values increased to approximately 12.9 regardless of retention time. Previous study [29] demonstrated that, the leaching behavior as a function of pH was observed with Pb, and the minimum solubility was obtained when the pH of leachate was close to 9. Distinctly, the pH level in this study was adverse to lowering leachability of Pb. While, the mobility of Pb indeed decreased in Fig 3, implying that the efficiency of method in this study was less susceptible to pH value of leachate.

### 3.3. Effect of sodium hydroxide addition

**Fig 4:**
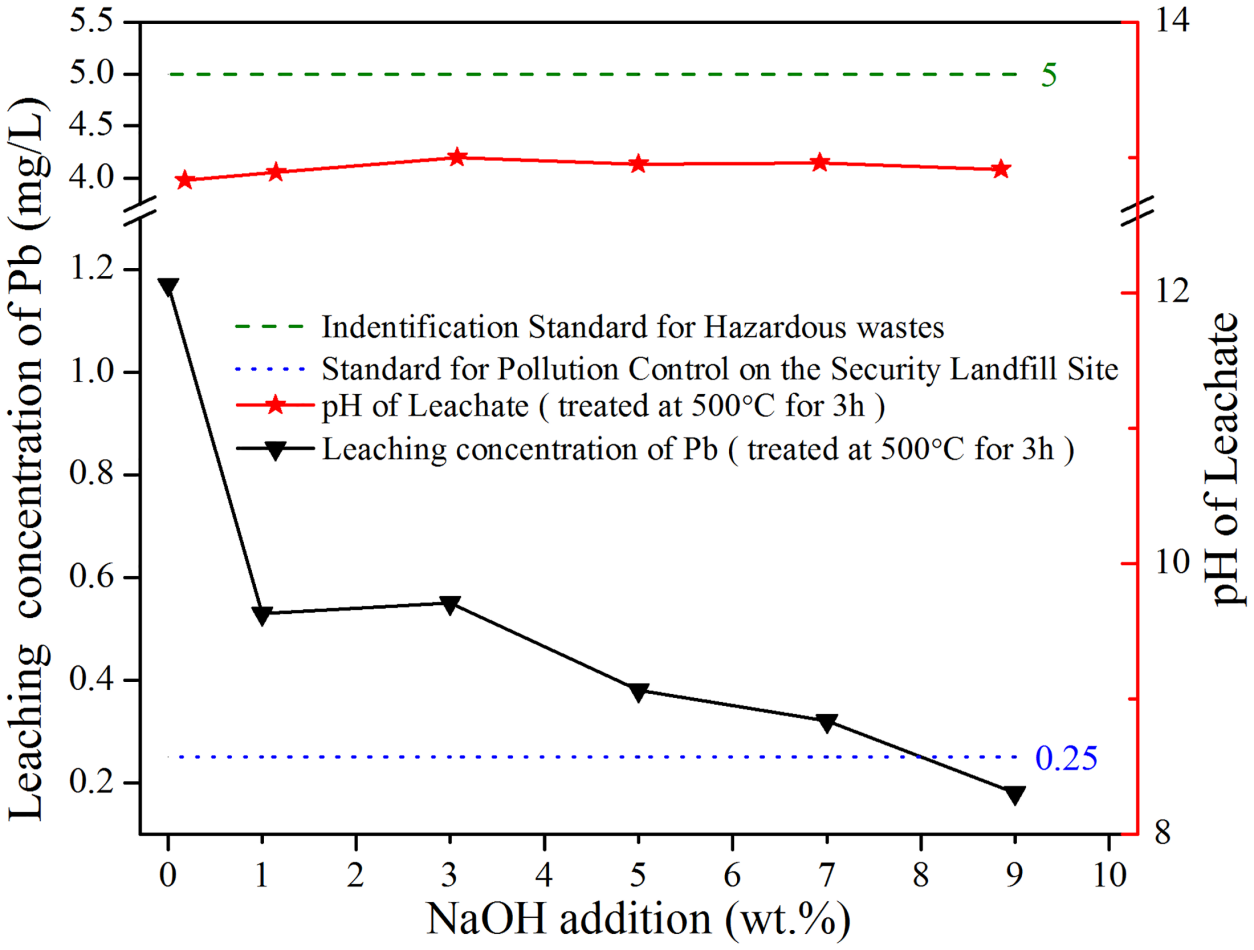
TCLP leaching concentrations of Pb as a function of the amount of NaOH addition.

To study the influence of NaOH addition on leaching of Pb, the samples with different amounts of NaOH were thermal treated at 500°C for 3 h, respectively. Leaching concentrations of Pb from treated residues and pH of leachate as a function of NaOH addition are reported in Fig 4. With or without NaOH, leaching concentrations of Pb in all treated samples were below the limit in the S1 (5 mg/L). During thermal process, different IFA particles agglomerated to generate relatively compact particle clusters resulting in impairing leaching of Pb. Furthermore, it could be seen that leaching values of Pb dramatically decreased with the addition of NaOH, and the larger amount of NaOH was added, lower Pb concentration was realized. As discussed in 3.2, with NaOH introduced, conglomeration occurred more easily due to its properties of hydroscopicity and low melting point (318°C). At the same time, complex reactions like carbonation, hydrothermal process in microcell happened in heating course and restrained leaching behavior of Pb in treated products. Particularly, when mass proportion of NaOH rose up to 9%, leaching concentration of Pb decreased to 0.18 mg/L below the rigorous limitation in S2 (0.25 mg/L). These experimental results manifested that, the optimum mass proportion of NaOH was 9%. From Fig 4 and S2 Table, we could see that pH values of all leachates remained in the same level around 12.9, which was obedient to the results of R519, R529, R539.

### 3.4. Phase composition of raw and thermal treated IFAs

**Fig 5:**
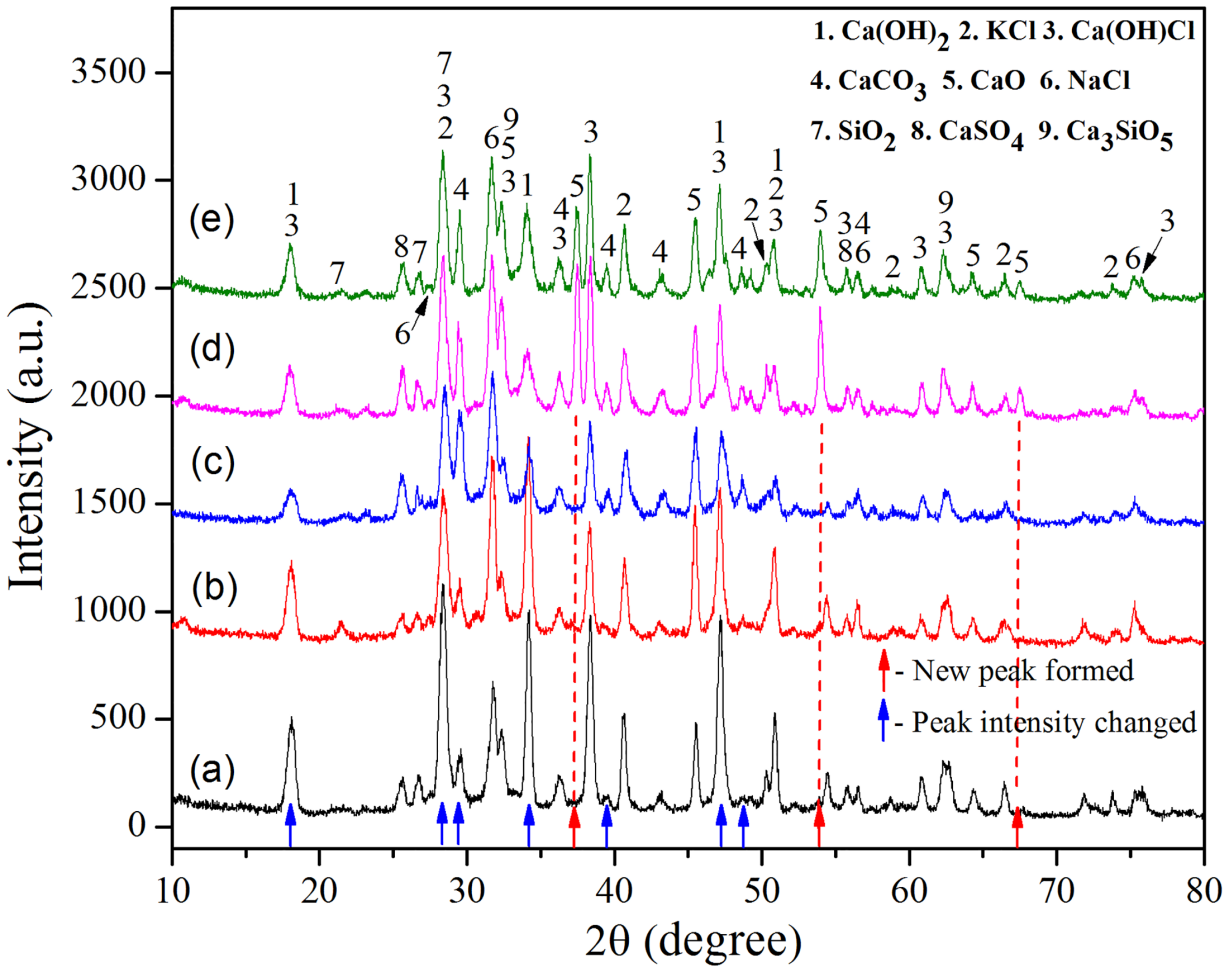
XRD pattern of (a) raw IFA; (b) R319; (c) R439; (d) R519, F; (e) R539.

In current work, XRD was employed to provide detailed information for phase alteration before and after thermal treatment. Fig 5 depicts XRD patterns of raw and treated residues under different temperatures and retention times. The main compounds, identified in raw IFA, were KCl, NaCl, Ca(OH)_2_, Ca(OH)Cl, CaCO_3_ and SiO_2_. It was reported that, due to the existence of chlorine, when temperature was above 800°C heavy metal chlorides were easily formed and vaporized [26]. Vaporization of chlorides went against immobilizing Pb through partly loosening the particle structure. In this study, however, the highest operating temperature was only 500°C, and therefore vaporization of heavy metals could be ignored. In the MSW incineration plant, lime slurry was injected into flue gas system for air pollution control, which was the most important explanation for the existence of Ca(OH)_2_ in IFA. Moreover, in APC system, CO_2_ in flue gas was easily to react with Ca(OH)_2_ forming CaCO_3_. Similarly, weathering was another crucial factor that facilitated the transformation of Ca(OH)_2_ to CaCO_3_ when the IFA pile was exposed to circumstance. As for Ca(OH)Cl, it was produced by HCl absorption in the following way [30]:

$$Ca{\left(OH\right)}_{2}+HCl\to Ca\left(OH\right)Cl+H_{2}O$$ (1)

As pointed in Fig 5 by blue arrows, it could be noticed that the relative intensities of Ca(OH)_2_, Ca(OH)Cl, CaCO_3_ peaks changed a lot, but compound species did not transform in products treated below 500°C irrespective of durations. In particular, the relative intensities of Ca(OH)_2_ and Ca(OH)Cl peaks decreased distinctly, while that of CaCO_3_ was improved. Besides compositions were identified in raw ash, and CaO also appeared in residues treated at 500°C marked in Fig 5 with red arrows. This indicated that only when operating temperature was 500°C high or higher temperature, CaO will only be formed in the system. In leaching procedure, CaO reacted with water to generate Ca(OH)_2_, and it promoted the pH values of leachates, which was proved from the results in Figs 3 and 4 that pH values of leachate of residues detoxified at 500°C were in a higher level than that of residues treated at 300°C and 400°C. These changes were likely to be caused as follows [31]:

$$Ca{\left(OH\right)}_{2}+CO_{2}\to CaCO_{3}+H_{2}O$$ (2)

$$Ca{\left(OH\right)}_{2}\to CaO+H_{2}O$$ (3)

Ca(OH)Cl→CaO+HCl (4)

Among these changes, Eq (2) was a process of carbonation. In this process, encapsulation and adsorption happened simultaneously as CaCO_3_ was generated [13]. These physical and chemical alterations in the system successfully suppressed leaching of Pb [32]. In the present study, the addition of NaOH had little influence on the pH values of leachates of fly ash. Because NaOH reacted with silica and alumina forming aluminosilicate mineral, and these products did not significantly change the pH values of leachates. Actually, CaO, according to above explanations, mainly regulated the alteration of pH values of leachates [33]. However, the generation of CaO was not affected by the addition of NaOH, so the pH values of the leachates were almost same even for that without the addition of NaOH.

S1 Fig shows XRD patterns of raw IFA and residues treated at 500°C for 3 h with different amounts of NaOH addition. Obviously, with or without NaOH addition, the relative intensities of Ca(OH)_2_ and Ca(OH)Cl peaks reduced, and CaO peaks emerged. Although the amount of the added NaOH was improved from 0 to 9%, there was little deviation in compositions of residues treated at 500°C, which was in agreement with results in Fig 5. It was noteworthy that NaOH or its possible transformations like Na_2_CO_3_ and sodium aluminosilicate were not detected by XRD in both Fig 5 and S1 Fig. This might be because NaOH reacted with CO_2_ or SiO_2_ and Al_2_O_3_ to form amorphous carbonates and/or aluminosilicates rather than crystalline phase [34, 35].

### 3.5. Morphology of raw and thermal treated IFAs

**Fig 6:**
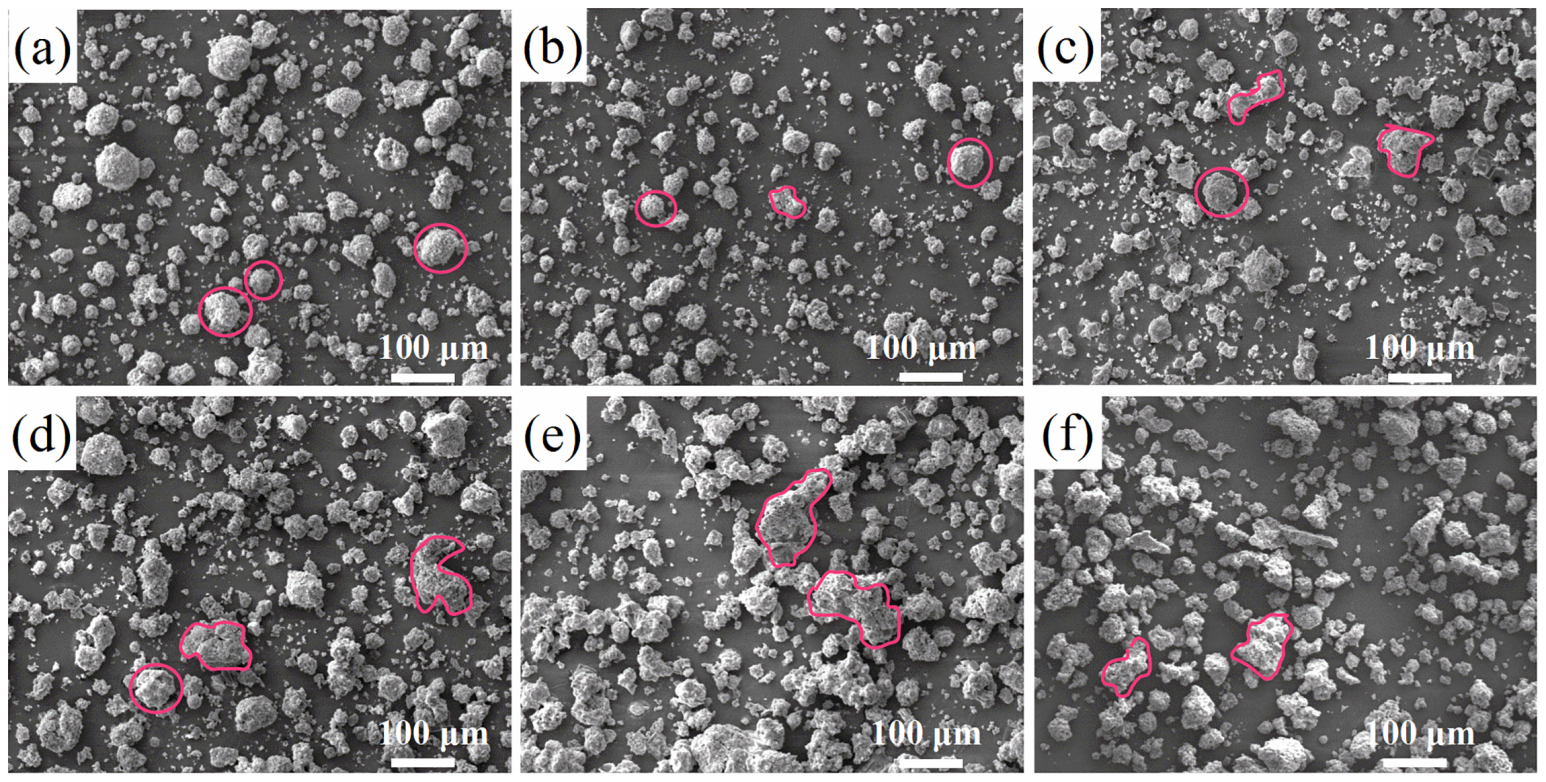
SEM images of (a) raw IFA; (b) R319,; (c) R339; (d) R439; (e) R519; (f) R539.

In this study, SEM-EDS analysis was conducted to characterize morphology and composition features of individual particles. Fig 6 presents the SEM graphs of fresh ash and thermal treated slags under different conditions. It was shown in Fig 6(a), that most particles in raw ash were of spherical shape. When processing temperature was 300°C, the number of spherical particles decreased and a few of irregular particles appeared in Fig 6(b). Besides, with increasing temperature and holding time, the enhancement of the number of irregular particles was apparent in Fig 6(c) and 6(d). As temperature rose up to 500°C, spherical particles were seldom found in Fig 6(e) and 6(f) indicating that at 500°C temperature, intensive particle integration occurred. As a result, the leaching concentrations of Pb in thermal treated residues were diminished. S2 Fig shows low-magnification SEM images of nascent IFA and residues processed at 500°C for 3 h with different amounts of NaOH addition. Similarly, treated particles were almost completely in roughly shaped forms.

**Fig 7:**
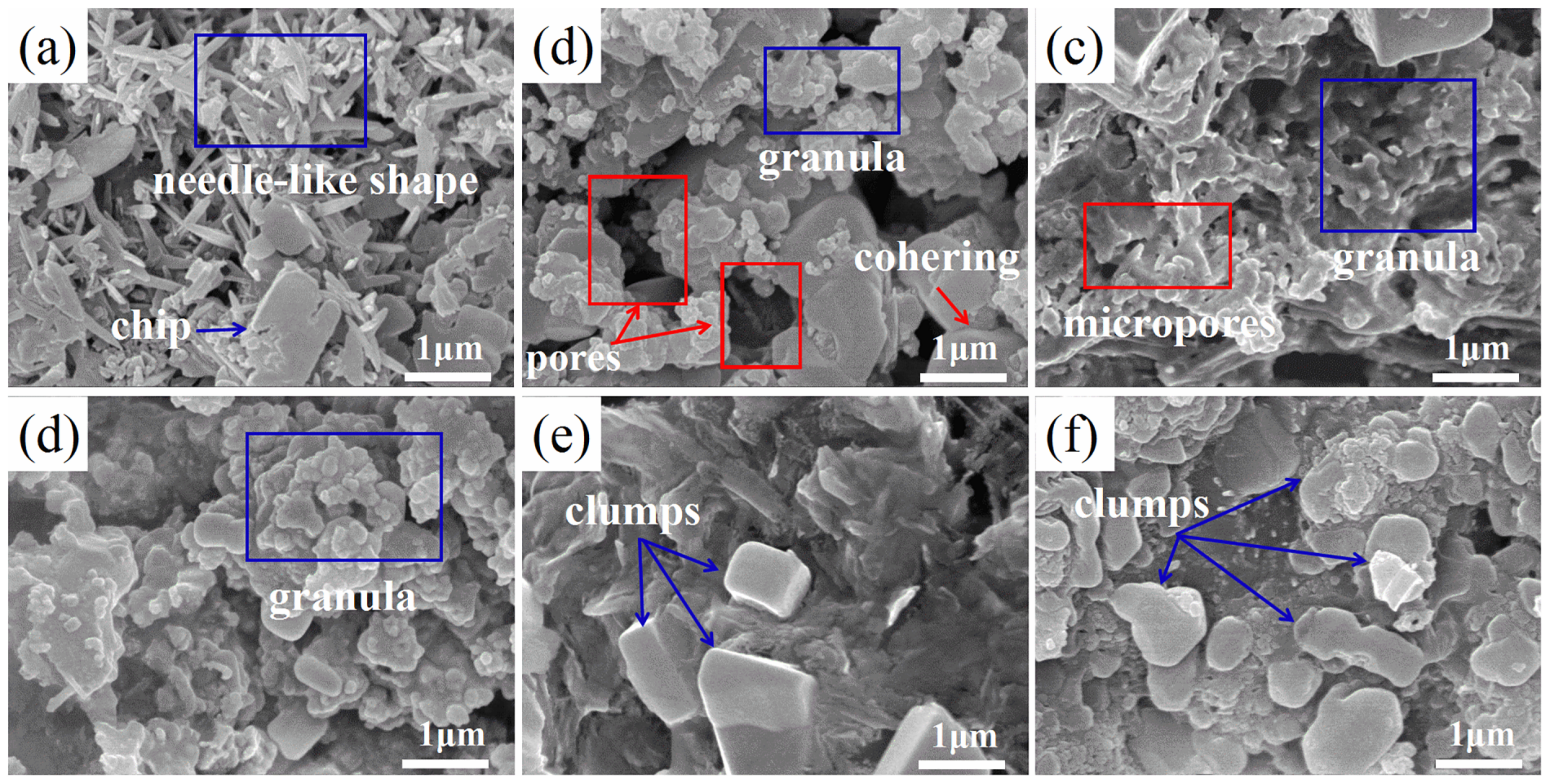
Microstructure images of (a) raw IFA; (b) R319; (c) R339; (d) R439; (e) R519; (f) R539.

Microstructures of raw IFA and these thermal products treated under various conditions are exhibited in Fig 7 and S3 Fig. In Fig 7(a), it was revealed that fresh ash was inhomogeneous in crystalline shapes and contained many needle-like and flake shaped crystallites. However, in Fig 7(b) needle shaped crystallites were scarcely present and there existed numerous granules. In raw IFA, large-sized pores could hardly be observed in particles of raw ash because the pores were covered by needle-like structure. To the contrary, it was noteworthy that some relatively larger pores appeared simultaneously after treatment. These results suggested that granules in Fig 7(b) were likely to originate from needle-like crystallites in Fig 7(a) by melting. Generally, because lathy bars had large specific area, they were easily fused. As a result, molten products covered the surface of particles and partly filled the large pores in the particles of raw ash, cutting down the leaching concentrations of Pb in treated samples. As shown in Fig 7(c) and 7(d), large pores were eliminated and substituted for micropores, which were beneficial for leaching reduction of Pb. As for specimens detoxified at 500°C, pores nearly vanished and granules aggregated into clumps marked in Fig 7(e) and 7(f). Thus, Pb was encapsulated into clumps, which resulted in further decreasing the concentrations of Pb.

In S3 Fig, the block was dominant shape of crystallites after treated at 500°C for 3 h with or without NaOH addition. The influence of the amount of added NaOH on microstructure was inconspicuous implying that more amounts of NaOH wakened leaching of Pb not predominantly by further densifying the structure. Practically, when NaOH was mixed with IFA, water-rich particle clusters were produced due to its strong water absorption. In heating process, hydrothermal solidification and carbonation in watery clusters happened, and confined the leaching of Pb. It was evident that more NaOH amount leaded to larger scale of hydrothermal solidification and carbonation, which made contributions to lower concentration of Pb. In addition, NaOH was prone to form liquid phase during heating course owing to its relatively low melting point (318°C). Accordingly, role of co-solvent played by NaOH and its corrosive property both did bad damages to original construction of particles facilitating particles coherence, clumps formation and densification. According to these mechanisms, Pb was entrapped inside, and newly formed large particle clusters or on the grain boundary, contributing to being hard to leach.

**Fig 8:**
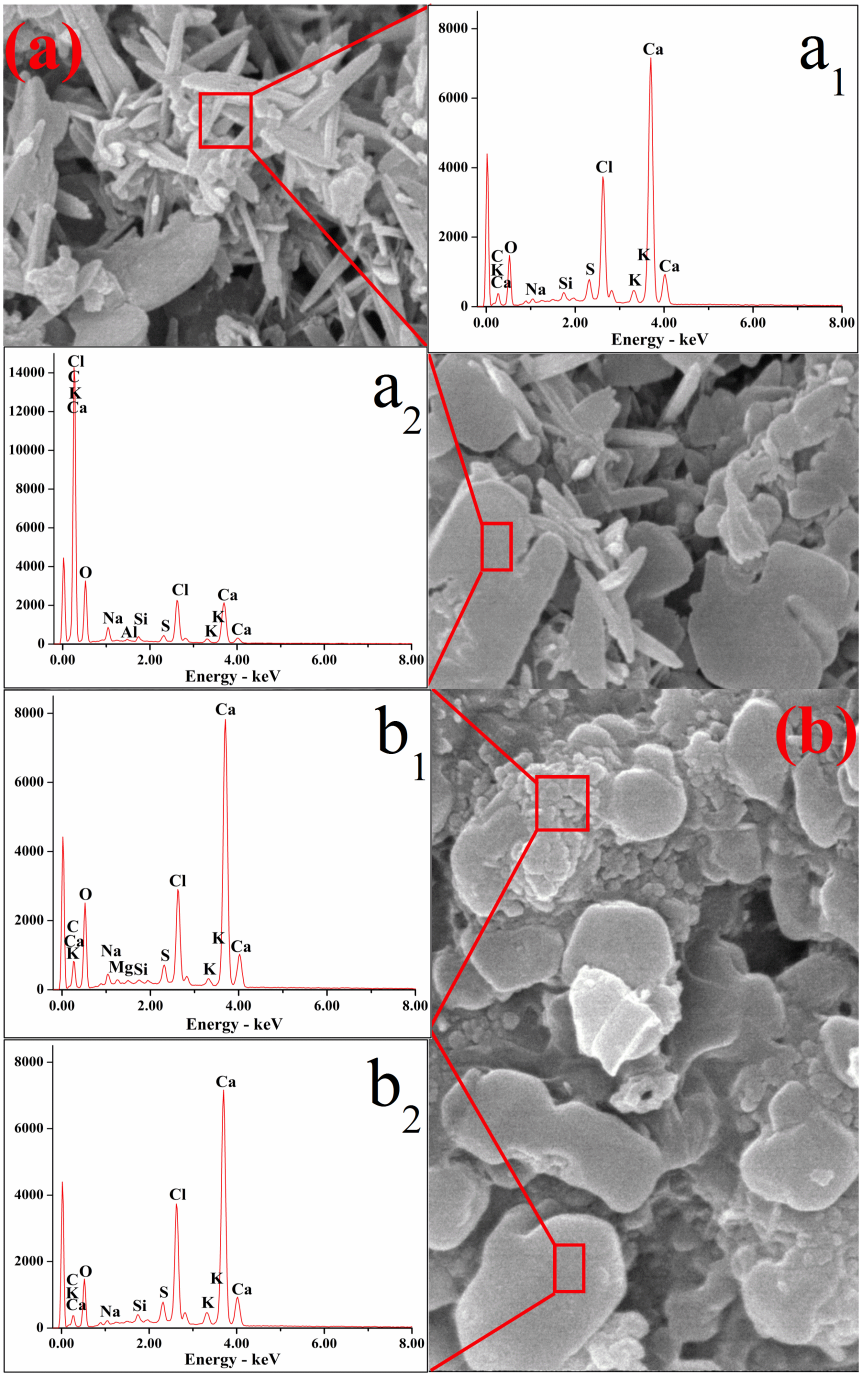
SEM and corresponding EDS images of (a) raw IFA: a_1_, composition of needle-like crystallites; a_2_, composition of flake-like crystallite; (b) R539: b_1_, composition of granules; b_2_, composition of clump.

The results of EDS analysis for typical microstructure of IFA particles are shown in Fig 8 and S3 Table. The EDS outcomes indicated that the elemental compositions of the surface of crystallites in different shapes were varying, which was constant with earlier studies [26]. As shown in Fig 8(a)-a_1_ and S3 Table, the needle shaped crystallites were characteristic of high contents of C, O, Cl and Ca, implying the existence of CaCO_3_, Ca(OH)Cl and Ca(OH)_2_. In Fig 8(a)-a_2_ and S3 Table, it was observed that flake-like materials were rich in C and O reaching to 93.32% in total, implying the presence of large amounts of carbonates. As for elemental compositions of granular and clump shaped crystallites, the former contained large amounts of Na and Cl, indicating the existences of NaCl and other chlorides, while the later were abundant in C, O, Cl and Ca which were similar to the compositions of needle-like crystallites. It was important to highlight that Pb and other heavy metals were not detected, which, to some degree, demonstrated that most heavy metals were present inside the particles or were adsorbed in holes or hollows. Consequently, when size of holes decreased or the holes were sealed, leaching concentrations of Pb in treated residues fell off remarkably in Figs 3 and 4.

### 3.6. Distribution of Pb pattern

**Fig 9:**
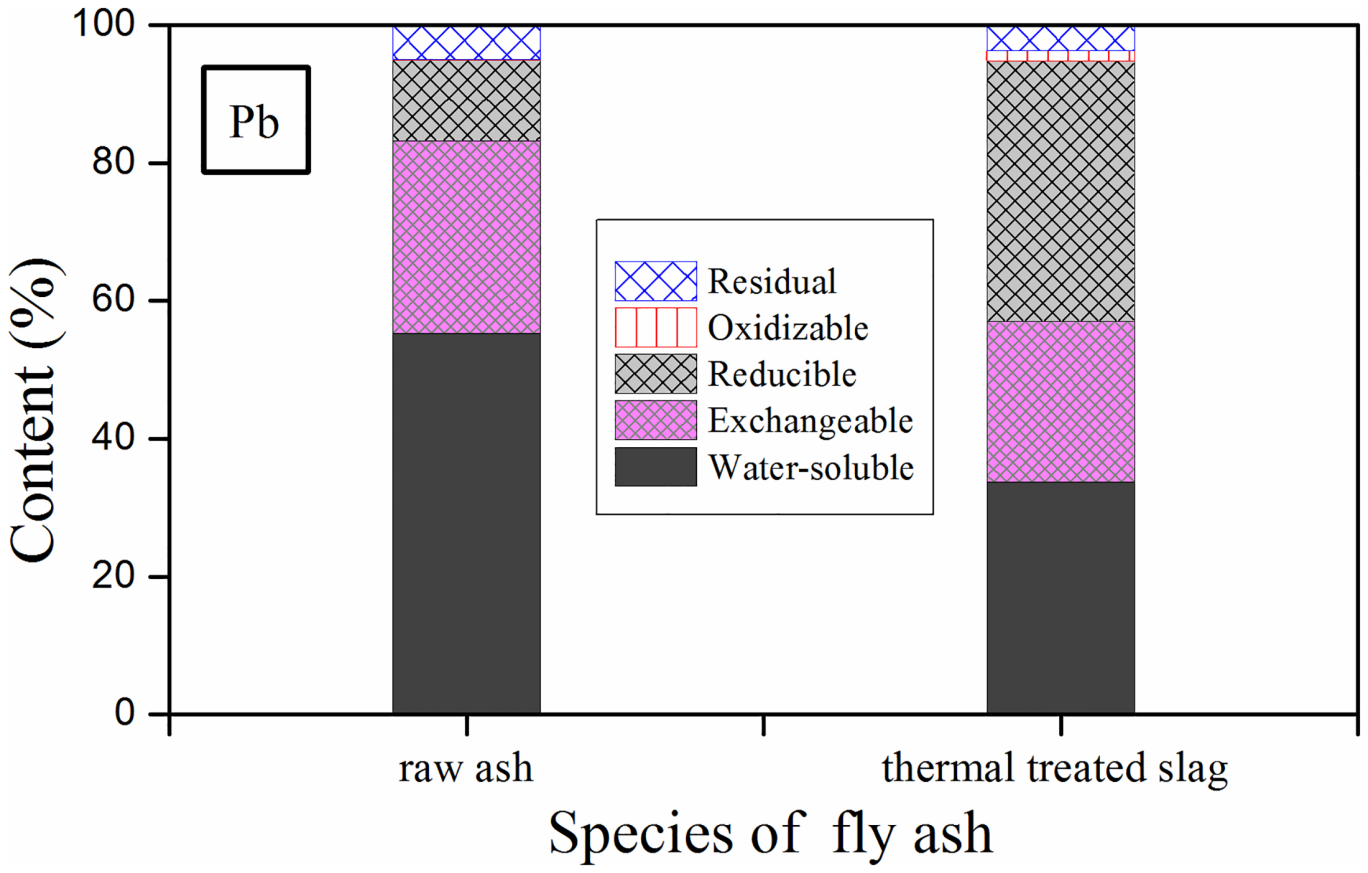
Distribution pattern of Pb in (a) raw IFA; (b) R539.

A modified version of sequential extraction procedure was involved for examining the distribution of Pb before and after thermal treatment [36]. The experimental parameters are summarized in Table 4, and the result is plotted in Fig 9.

**Table 4:** Experimental conditions of sequential extraction procedure.

Speciation	Extraction agent	Mixing time (h)	Temperature (°C)
Water-soluble	40 mL deionized water (pH = 4.0)	16	25
Exchangeable	40 mL 0.1 M CH_3_COOH (pH = 2.9)	16	25
Easily-reduced	40 mL 0.1M NH_2_OH-HCl (pH = 2.0)	16	25
Oxidizable-step1	10 mL 30% H_2_O_2_	1	50
Oxidizable-step2	10 mL 30% H_2_O_2_	1	50
Oxidizable-step3	50 mL 1M CH_3_COONH_4_	16	25
Residual-step1	20 mL HF	2	50
Residual-step2	20 mL HF + 20 mL 65% HNO_3_	2	50
Residual-step3	20 mL 30% HCl	2	50

Comparing the speciation of Pb in nascent IFA and treated slag, big differences in distribution fractions of Pb were found. In raw IFA, water-soluble and exchangeable fractions accounted for 55.3% and 27.9%, respectively. The percentages of other three parts including easily-reduced, oxidizable and residual fractions were 11.8%, 0.06%, and 5.0%, separately. In thermal treated slag, however, the percentage of water-soluble fraction remarkably decreased to 33.8%, and that of exchangeable fraction descended to 23.3%. To the contrary, the ratio of easily-reduced fraction in Pb in thermal product was promoted to 37.8%. In general, many researchers held the viewpoint that water-soluble and exchangeable fractions were the most easily-extracted parts under natural conditions, and therefore the two fractions posed a huge threat to our environment [37, 38]. Through thermal process in this study, percentage of water-soluble plus exchangeable fractions totally dropped by 26% indicating that the distribution of Pb in MSW IFA was converted to relatively stable fractions, and possible hazardous effects of fly ash on surroundings caused by the leaching of Pb were significantly diminished.

## Conclusion

Due to the thriving development of MSW incineration, there will be increasingly large amounts of MSW IFA to be managed. Therefore, feasible treatment methods must be applied to detoxify IFA. In this study, a moderate thermal treatment with NaOH addition was adopted to stabilize Pb with increasing temperature from 300 to 500°C. TCLP leaching concentrations of Pb in thermal treated IFAs were markedly reduced to below the regulation of the identification standard for hazardous wastes in China (5 mg/L). With increases of temperature, retention time and amount of NaOH addition, leaching concentrations of Pb were further reduced. The optimum temperature was 500°C, retention time 3 h and the amount of NaOH addition 9%. Under the condition, leaching concentration of Pb was lowered to 0.18mg/L which was below the strict regulation of the standard for pollution control on the security landfill site in China (0.25 mg/L). After thermal process, the contents of Ca(OH)_2_, Ca(OH)Cl decreased, and that of CaCO_3_ increased. In particular, when it was up to 500°C, CaO was newly produced. Intensive agglomeration happened and great changes took place in shapes of crystallites during thermal treatment. Specifically, outline of particles changed from spherical type to irregular type and needle-like crystallites were substituted with granule and clump shaped crystallites. EDS results manifested that enrichment characteristic of elements in various shaped crystallites was different. According to sequential extraction procedure, in raw IFA, the two main fractions of Pb were water-soluble and exchangeable fractions accounting for 55.3% and 27.9%, respectively. While in thermal treated residues, the percentages of the two fractions reduced to 33.8% and 23.3%, which indicated that Pb was less leachable after thermal treatment. Therefore, this work provides an extremely simple but effective treatment process for IFA under relative low temperature without any pre-treatment or post-treatment.

## Supporting information

S1 Fig XRD pattern of (a) raw IFA; (b) R530 (c) R531; (d) R535; (e) R539. (DOCX)

S2 Fig SEM images of (a) raw IFA; (b) R530; (c) R531; (d) R535; (e) R539. (DOCX)

S3 Fig Microstructure images of (a) raw IFA; (b) R530; (c) R531; (d) R535; (e) R539. (DOCX)

S1 Table pH values of leachates for different treated residues. (DOCX)

S2 Table pH values of leachates for thermal treated slags with different amounts of NaOH addition. (DOCX)

S3 Table Main elemental components of different microstructures in raw and treated IFA (wt. %). (DOCX)

## References

[1] Ma J, Hipel KW. Exploring social dimensions of municipal solid waste management around the globe—a systematic literature review. Waste Manag. 2016; 56: 3–12. 10.1016/j.wasman.2016.06.041 27406309

[2] Liu Y, Wang H, Weng S, Su W, Wang X, Guo Y, et al Occupational hearing loss among Chinese municipal solid waste landfill workers: a cross-sectional study. PloS One. 2015; 10(6): e0128719 10.1371/journal.pone.0128719 26042421 PMC4455999

[3] Gong Y, Tian H, Wang L, Yu S, Ru S. An integrated approach combining chemical analysis and an in vivo bioassay to assess the estrogenic potency of a municipal solid waste landfill leachate in Qingdao. PloS One. 2014; 9(4): e95597 10.1371/journal.pone.0095597 24743634 PMC3990707

[4] Jin Y, Ma X, Jiang X, Liu H, Li X, Yan J, et al Effects of hydrothermal treatment on the major heavy metals in fly ash from municipal solid waste incineration. Energy Fuels. 2012; 27(1): 394–400.

[5] Sabbas T, Polettini A, Pomi R, Astrup T, Hjelmar O, Mostbauer P, et al Management of municipal solid waste incineration residues. Waste Manag. 2003; 23(1): 61–88. 10.1016/S0956-053X(02)00161-7 12623102

[6] Sakai S, Sawell SE, Chandler AJ, Eighmy TT, Kosson DS, Vehlow J, et al World trends in municipal solid waste management. Waste Manag. 1996; 16(5–6): 341–350.

[7] Min L, Xiang J, Song H, Sun LS, Sheng S, Li PS, et al Characterization of solid residues from municipal solid waste incinerator. Fuel. 2004; 83(10): 1397–1405.

[8] Ferreira C, Ribeiro A, Ottosen L. Possible applications for municipal solid waste fly ash. J Hazard Mater. 2003; B96(2–3): 201–216. 10.1016/s0304-3894(02)00201-7 12493209

[9] Le Forestier Lydie, Libourel G. Characterization of flue gas residues from municipal solid Waste combustors. Environ Sci Technol. 1998; 32(15): 2250–2256. 10.1021/es048693x 15871232

[10] Katsuura H, Inoue T, Hiraoka M, Sakai S. Full-scale plant study on fly ash treatment by the acid extraction process. Waste Manag. 1996; 16(5–6): 491–499.

[11] Margallo M, Taddei MBM, Hernández-Pellón A, Aldaco R, Irabien Á. Environmental sustainability assessment of the management of municipal solid waste incineration residues: a review of the current situation. Clean Technol Envir. 2015; 17(5): 1333–1353.

[12] Chi M. Effects of modulus ratio and dosage of alkali-activated solution on the properties and micro-structural characteristics of alkali-activated fly ash mortars. Constr Build Mater. 2015; 99: 128–136.

[13] Jiang J, Chen M, Zhang Y, Xu X. Pb stabilization in fresh fly ash from municipal solid waste incinerator using accelerated carbonation technology. J Hazard Mater. 2009; 161(2–3): 1046–51. 10.1016/j.jhazmat.2008.04.051 18502039

[14] Quina MJ, Bordado JC, Quinta-Ferreira RM. Treatment and use of air pollution control residues from MSW incineration: an overview. Waste Manag. 2008; 28(11): 2097–2121. 10.1016/j.wasman.2007.08.030 18037284

[15] Astrup T. Pretreatment and utilization of waste incineration bottom ashes: Danish experiences. Waste Manag. 2007; 27(10): 1452–1457. 10.1016/j.wasman.2007.03.017 17512719

[16] Todorovic J, Ecke H. Demobilisation of critical contaminants in four typical waste-to-energy ashes by carbonation. Waste Manag. 2006; 26(4): 430–441. 10.1016/j.wasman.2005.11.011 16403618

[17] Wey MY, Liu KY, Tsai TH, Chou JT. Thermal treatment of the fly ash from municipal solid waste incinerator with rotary kiln. J Hazard Mater. 2006; 137(2): 981–989. 10.1016/j.jhazmat.2006.03.024 16647203

[18] Wang Q, Yan J, Tu X, Chi Y, Li X, Lu S, et al Thermal treatment of municipal solid waste incinerator fly ash using DC double arc argon plasma. Fuel. 2009; 88(5): 955–958.

[19] Zhang H, Zhao Y, Qi J. Study on use of MSWI fly ash in ceramic tile. J Hazard Mater. 2007; 141(1): 106–114. 10.1016/j.jhazmat.2006.06.100 16889889

[20] Polettini A, Pomi R, Trinci L, Muntoni A, Lo MS. Engineering and environmental properties of thermally treated mixtures containing MSWI fly ash and low-cost additives. Chemosphere. 2004; 56(10): 901–910. 10.1016/j.chemosphere.2004.05.004 15268956

[21] Lin CF, Wu CH, Ho HM. Recovery of municipal waste incineration bottom ash and water treatment sludge to water permeable pavement materials. Waste Manag. 2006; 26(9): 970–8. 10.1016/j.wasman.2005.09.014 16293405

[22] Sakai SI, Hiraoka M. Municipal solid waste incinerator residue recycling by thermal processes. Waste Manag. 2000; 20(2–3): 249–258.

[23] Lindberg D, Molin C, Hupa M. Thermal treatment of solid residues from WtE units: A review. Waste Manag. 2015; 37: 82–94. 10.1016/j.wasman.2014.12.009 25592372

[24] Lombardi L, Carnevale E, Corti A. A review of technologies and performances of thermal treatment systems for energy recovery from waste. Waste Manag. 2014; 37: 26–44. 10.1016/j.wasman.2014.11.010 25535103

[25] Wu MH, Lin CL, Zeng WY. Effect of waste incineration and gasification processes on heavy metal distribution. Fuel Process Technol. 2014; 125(125): 67–72.

[26] Yu J, Qiao Y, Jin L, Ma C, Paterson N, Sun L. Removal of toxic and alkali/alkaline earth metals during co-thermal treatment of two types of MSWI fly ashes in China. Waste Manag. 2015; 46: 287–297. 10.1016/j.wasman.2015.08.005 26303652

[27] EPA C. Solid waste-extraction procedure for leaching toxicity-acetic aid buffer solution method. HJ/T 300–2007.

[28] Hu Y, Zhang P, Li J, Chen D. Stabilization and separation of heavy metals in incineration fly ash during the hydrothermal treatment process. J Hazard Mater. 2015; 299(4): 149–157. 26100935 10.1016/j.jhazmat.2015.06.002

[29] Quina MJ, Bordado JC, Quinta-Ferreira RM. The influence of pH on the leaching behaviour of inorganic components from municipal solid waste APC residues. Waste Manag. 2009; 29(9): 2483–2493. 10.1016/j.wasman.2009.05.012 19545989

[30] Bausach M, Krammer G, Cunill F. Reaction of Ca(OH)_2_ with HCl in the presence of water vapour at low temperatures. Thermochim Acta. 2004; 421(1–2): 217–223.

[31] Quina MJ, Almeida MA, Santos R, Quinta-Ferreira RM, Bordado JM. Compatibility analysis of municipal solid waste incineration residues and clay for producing lightweight aggregates. Appl Clay Sci. 2014; 102(102): 71–80.

[32] Ecke H, Menad N, Lagerkvist A. Carbonation of municipal solid waste incineration fly ash and the impact on metal mobility. J Environ Eng. 2003; 129(5): 435–440.

[33] Hu HY, Liu H, Shen WQ, Luo GQ, Li AJ, Lu ZL, et al Comparison of CaO's effect on the fate of heavy metals during thermal treatment of two typical types of MSWI fly ashes in China. Chemosphere. 2013; 93(4): 590–596. 10.1016/j.chemosphere.2013.05.077 23800595

[34] Zhu F, Takaoka M, Oshita K, Takeda N. Comparison of two types of municipal solid waste incinerator fly ashes with different alkaline reagents in washing experiments. Waste Manag. 2009; 29(1): 259–264. 10.1016/j.wasman.2008.03.008 18539449

[35] Zheng L, Wang W, Shi Y. The effects of alkaline dosage and Si/Al ratio on the immobilization of heavy metals in municipal solid waste incineration fly ash-based geopolymer. Chemosphere. 2010; 79(6): 665–671. 10.1016/j.chemosphere.2010.02.018 20304461

[36] Nurmesniemi H, Pöykiö R, Kuokkanen T, Rämö J. Chemical sequential extraction of heavy metals and sulphur in bottom ash and in fly ash from a pulp and paper mill complex. Waste Manag Res. 2008; 26(4): 389–399. 10.1177/0734242X07079051 18727331

[37] Chou JD, Wey MY, Chang SH. Evaluation of the distribution patterns of Pb, Cu and Cd from MSWI fly ash during thermal treatment by sequential extraction procedure. J Hazard Mater. 2008; 162(2–3): 1000–1006. 10.1016/j.jhazmat.2008.05.155 18614278

[38] Yliniemi J, Pesonen J, Tiainen M, Illikainen M. Alkali activation of recovered fuel-biofuel fly ash from fluidised-bed combustion: stabilisation/solidification of heavy metals. Waste Manag. 2015; 43(5): 273–282. 26054963 10.1016/j.wasman.2015.05.019

