# Supplementary material for: Stabilization of lead in incineration fly ash by moderate thermal treatment with sodium hydroxide addition
Source: PLoS One. 2017 Jun 6;12(6):e0178816. doi: 10.1371/journal.pone.0178816 (PMC5460817; doi:10.1371/journal.pone.0178816)
Supplement: S3 Fig — (DOCX) [file pone.0178816.s003.docx]

**S3 Fig.** Microstructure images of (a) raw fly ash; (b) R530; (c) R531; (d) R535; (e) R539.
