# Supplementary material for: Stabilization of lead in incineration fly ash by moderate thermal treatment with sodium hydroxide addition
Source: PLoS One. 2017 Jun 6;12(6):e0178816. doi: 10.1371/journal.pone.0178816 (PMC5460817; doi:10.1371/journal.pone.0178816)
Supplement: S1 Table — (DOCX) [file pone.0178816.s004.docx]

**S1 Table** pH values of leachates of different treated residues.

| Sample name | Raw ash | R319 | R329 | R339 | R419 | R429 | R439 | R 519 | | R529 | | R529 | |
| --- | --- | --- | --- | --- | --- | --- | --- | --- | --- | --- | --- | --- | --- |
| pH | 11.94 | 12.15 | 12.17 | 12.14 | 12.30 | 12.20 | 12.18 | 12.85 | 12.97 | | 12.91 | |  |
