# Supplementary material for: Stabilization of lead in incineration fly ash by moderate thermal treatment with sodium hydroxide addition
Source: PLoS One. 2017 Jun 6;12(6):e0178816. doi: 10.1371/journal.pone.0178816 (PMC5460817; doi:10.1371/journal.pone.0178816)
Supplement: S2 Table — (DOCX) [file pone.0178816.s005.docx]

**S2 Table** pH values of leachates of thermal treated slag with different amount of NaOH addition.

| Sample name | R530 | R531 | R533 | R535 | R537 | R539 |
| --- | --- | --- | --- | --- | --- | --- |
| pH | 12.83 | 12.89 | 13.00 | 12.95 | 12.96 | 12.91 |
