# Supplementary material for: Stabilization of lead in incineration fly ash by moderate thermal treatment with sodium hydroxide addition
Source: PLoS One. 2017 Jun 6;12(6):e0178816. doi: 10.1371/journal.pone.0178816 (PMC5460817; doi:10.1371/journal.pone.0178816)
Supplement: S3 Table — (DOCX) [file pone.0178816.s006.docx]

**S3 Table** Main elemental components of different microstructures in raw and treated fly ashes (wt.%).

| Element  Image | C K | O K | Na K | Si K | S K | Cl K | K K | Ca K |
| --- | --- | --- | --- | --- | --- | --- | --- | --- |
| a_1_ | 13.68 | 41.05 | 0.73 | 0.61 | 1.64 | 11.45 | 1.37 | 29.47 |
| a_2_ | 66.58 | 26.74 | 0.96 | 0.18 | 0.28 | 2.23 | 0.23 | 2.74 |
| b_1_ | 0 | 0 | 28.04 | 0 | 0.62 | 61.87 | 0.97 | 8.50 |
| b_2_ | 18.41 | 46.69 | 1.31 | 0.19 | 1.15 | 6.73 | 0.55 | 24.27 |
